# 6-Chloro-*N* ^4^-methyl-*N* ^4^-phenyl­pyrimidine-4,5-diamine

**DOI:** 10.1107/S1600536811028303

**Published:** 2011-07-23

**Authors:** Fuqiang Shi, Li-Hong Zhu, Long Zhang, Ya-Feng Li

**Affiliations:** a School of Chemical Engineering, Changchun University of Technology, Changchun 130012, People’s Republic of China

## Abstract

In the title compound, C_11_H_11_ClN_4_, the dihedral angle between the aromatic rings is 66.47 (8)°. In the crystal, mol­ecules are linked by N—H⋯N hydrogen bonds, generating *C*(5) chains propagating in [010]. Slipped aromatic π–π stacking between centrosymmetrically related pairs of pyrim­idine rings also occurs [centroid–centroid separation = 3.7634 (12)Å and slippage = 1.715 Å].

## Related literature

For background to pyrimidines, see: Barillari *et al.* (2001 ▶); Gangjee *et al.* (2010 ▶). For slipped π–π stacking inter­actions, see: Glówka *et al.* (1999 ▶).
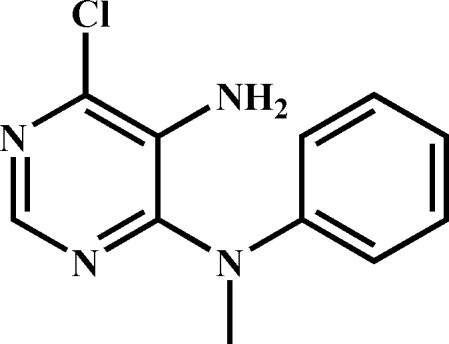


## Experimental

### 

#### Crystal data

C_11_H_11_ClN_4_
*M* *_r_* = 234.69
Monoclinic, 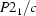

*a* = 9.5887 (19) Å
*b* = 9.948 (2) Å
*c* = 12.671 (3) Å
β = 109.63 (3)°
*V* = 1138.4 (4) Å^3^
*Z* = 4
Mo *K*α radiation
μ = 0.31 mm^−1^
*T* = 293 K
0.45 × 0.36 × 0.33 mm

#### Data collection

Rigaku R-AXIS RAPID diffractometer
Absorption correction: multi-scan (*ABSCOR*; Higashi, 1995 ▶) *T*_min_ = 0.872, *T*_max_ = 0.905
10835 measured reflections
2588 independent reflections
1983 reflections with *I* > 2σ(*I*)
*R*_int_ = 0.025

#### Refinement

*R*[*F* ^2^ > 2σ(*F* ^2^)] = 0.037
*wR*(*F* ^2^) = 0.105
*S* = 1.07
2588 reflections
146 parameters
H-atom parameters constrained
Δρ_max_ = 0.17 e Å^−3^
Δρ_min_ = −0.34 e Å^−3^

### 

Data collection: *PROCESS-AUTO* (Rigaku, 1998) ▶; cell refinement: *PROCESS-AUTO* ▶; data reduction: *CrystalStructure* (Rigaku, 2002) ▶; program(s) used to solve structure: *SHELXS97* (Sheldrick, 2008 ▶); program(s) used to refine structure: *SHELXL97* (Sheldrick, 2008 ▶); molecular graphics: *DIAMOND* (Brandenburg, 2000 ▶); software used to prepare material for publication: *SHELXL97*.

## Supplementary Material

Crystal structure: contains datablock(s) I, global. DOI: 10.1107/S1600536811028303/hb5937sup1.cif

Structure factors: contains datablock(s) I. DOI: 10.1107/S1600536811028303/hb5937Isup2.hkl

Supplementary material file. DOI: 10.1107/S1600536811028303/hb5937Isup3.cml

Additional supplementary materials:  crystallographic information; 3D view; checkCIF report

## Figures and Tables

**Table 1:** Hydrogen-bond geometry (Å, °)

*D*—H⋯*A*	*D*—H	H⋯*A*	*D*⋯*A*	*D*—H⋯*A*
N4—H4*A*⋯N1^i^	0.86	2.28	3.0993 (18)	159

Symmetry code: (i) 
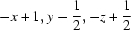
.

**Fig. 1.**
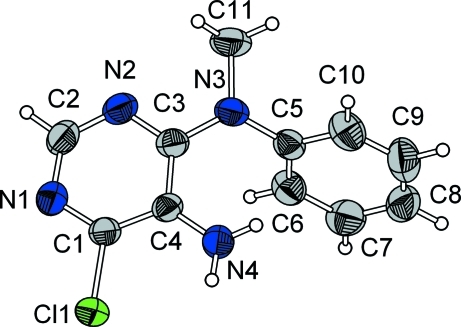
The title compound with displacement ellipsoids shown at the 50% probability level.

**Fig. 2.**
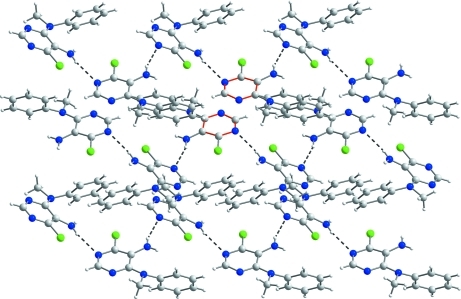
The hydrogen bond of amino group of pyrimidyl ring and the nitrogen of the adjacent pyrimidyl ring and π–π stacking of adjacent pyrimidyl rings.

## supplementary crystallographic information

### Comment

Pyrimidine diamines exhibit a wide range of biological activities (Barillari, *et al.*, 2001; Gangjee *et al.*,2010). Here, the crystal structure of the title compound, (I), is determined by X-ray single crystal diffraction.

In the structure of (I) (Fig. 1), *N*-methyl group links pyrimidyl and phenyl rings of which the dihedral angle is 66.62 (5)°. Two chloropyrimidyl rings of two adjacent molecules point to the opposite directions with *π*-*π* conjugation, in which stacking *h* (center-plane) is in 3.3411 Å, *d*(center-center) in 3.7633 Å and shift *r* (displacement of two centers) in 1.7319 Å (Glówka, *et al.*, 1999). The H-bond betwen amino group of pyrimidyl ring and the nitrogen of the adjacent pyrimidyl ring (N4—H4A···N1^i^) results in the formation of infinite chain (Fig. 2).

### Experimental

4,6-Dichloro-5-nitro-pyrimidine (5.20 g, 27 mmol), *N*-methylbenzenamine (3.2 mL, 32 mmol) and triethylamine (7.6 mL, 54 mmol) were dissolved in anhydrous THF (20 mL). The reaction mixture was stirred at room temperature overnight, concentrated *in vacuo*, diluted with water, and extracted with EtOAc. The organic phase was washed with 1mol/L HCl and brine, dried over anhydrous MgSO_4_, and concentrated *in vacuo* to give rise to the solid crude product. The recrystallization of crude product from methanol provided the desired pure product of 6-chloro-*N*-methyl-5-nitro-*N*- phenylpyrimidin-4-amine (yellow solid, 5.7g, 80% yield, m.p. 133.5-135.5 °C). 6-Chloro-*N*-methyl-5-nitro-*N*-phenylpyrimidin-4-amine (4.36g, 16.5 mmol) was dissolved in a mixture of ethanol (59.0 mL) and water (17.0 mL). Iron powder (2.8 g, 50mmol) and NH_4_Cl (0.56 g, 10.0 mmol) were added to it. The mixture was then stirred in reflux for 5 h, cooled to room temperature, and filtered through a pad of celite. The filtrate was concentrated *in vacuo*. The residue was extracted with EtOAc. The organic extract was washed with saturated NaHCO_3_, water, and brine and dried over anhydrous MgSO_4_. It was then filtered and concentrated *in vacuo* to the crude product which was purified by flash chromatography (elution with 9% EtOAc in petroleum ether followed by 20% EtOAc in petroleum ether) to give 6-chloro-*N*^4^-methyl-*N*^4^-phenylpyrimidine-4,5-diamine (white solid, 3.1g, 80% yield, m.p. 81.0-83.0 °C).

### Refinement

All H atoms were located from difference Fourier maps. H atoms attached to C atoms were treated as riding [C-H = 0.93-0.96 Å and Uiso(H) = 1.5Ueq(C) (methyl groups) or 1.2Ueq(C) (other H atoms)]. N-atom of amino group of pyrimidyl ring was treated as *sp*^2^ hybridization, and therefore H atoms of amino group were positioned as riding [N-H = 0.86 Å and Uiso(H) = 1.2Ueq(N)].

### Crystal data

**Table:**

C_11_H_11_ClN_4_	*F*(000) = 488
*M**_r_* = 234.69	*D*_x_ = 1.369 Mg m^−^^3^
Monoclinic, *P*2_1_/*c*	Mo *K*α radiation, λ = 0.71073 Å
Hall symbol: -P 2ybc	Cell parameters from 1000 reflections
*a* = 9.5887 (19) Å	θ = 3.1–27.5°
*b* = 9.948 (2) Å	µ = 0.31 mm^−^^1^
*c* = 12.671 (3) Å	*T* = 293 K
β = 109.63 (3)°	Block, colorless
*V* = 1138.4 (4) Å^3^	0.45 × 0.36 × 0.33 mm
*Z* = 4	

### Data collection

**Table:**

Rigaku R-AXIS RAPID diffractometer	2588 independent reflections
Radiation source: fine-focus sealed tube	1983 reflections with *I* > 2σ(*I*)
graphite	*R*_int_ = 0.025
Detector resolution: 10.00 pixels mm^-1^	θ_max_ = 27.5°, θ_min_ = 3.1°
ω scans	*h* = −12→12
Absorption correction: multi-scan (*ABSCOR*; Higashi, 1995)	*k* = −12→12
*T*_min_ = 0.872, *T*_max_ = 0.905	*l* = −16→16
10835 measured reflections	

### Refinement

**Table:**

Refinement on *F*^2^	Primary atom site location: structure-invariant direct methods
Least-squares matrix: full	Secondary atom site location: difference Fourier map
*R*[*F*^2^ > 2σ(*F*^2^)] = 0.037	Hydrogen site location: inferred from neighbouring sites
*wR*(*F*^2^) = 0.105	H-atom parameters constrained
*S* = 1.07	*w* = 1/[σ^2^(*F*_o_^2^) + (0.0596*P*)^2^ + 0.0777*P*] where *P* = (*F*_o_^2^ + 2*F*_c_^2^)/3
2588 reflections	(Δ/σ)_max_ < 0.001
146 parameters	Δρ_max_ = 0.17 e Å^−^^3^
0 restraints	Δρ_min_ = −0.34 e Å^−^^3^

### Special details

**Table:**

Geometry. All e.s.d.'s (except the e.s.d. in the dihedral angle between two l.s. planes) are estimated using the full covariance matrix. The cell e.s.d.'s are taken into account individually in the estimation of e.s.d.'s in distances, angles and torsion angles; correlations between e.s.d.'s in cell parameters are only used when they are defined by crystal symmetry. An approximate (isotropic) treatment of cell e.s.d.'s is used for estimating e.s.d.'s involving l.s. planes.
Refinement. Refinement of *F*^2^ against ALL reflections. The weighted *R*-factor *wR* and goodness of fit *S* are based on *F*^2^, conventional *R*-factors *R* are based on *F*, with *F* set to zero for negative *F*^2^. The threshold expression of *F*^2^ > σ(*F*^2^) is used only for calculating *R*-factors(gt) *etc*. and is not relevant to the choice of reflections for refinement. *R*-factors based on *F*^2^ are statistically about twice as large as those based on *F*, and *R*- factors based on ALL data will be even larger.

### Fractional atomic coordinates and isotropic or equivalent isotropic displacement parameters (Å^2^)

**Table:**

	*x*	*y*	*z*	*U*_iso_*/*U*_eq_	
Cl1	0.39218 (4)	0.36966 (4)	0.22870 (4)	0.06006 (16)	
N1	0.52906 (15)	0.54876 (12)	0.14917 (12)	0.0536 (3)	
N2	0.74328 (16)	0.50645 (12)	0.10019 (11)	0.0533 (3)	
N3	0.85704 (14)	0.29788 (13)	0.11914 (11)	0.0515 (3)	
N4	0.64945 (17)	0.20317 (13)	0.22608 (13)	0.0607 (4)	
H4A	0.5861	0.1794	0.2571	0.073*	
H4B	0.7179	0.1483	0.2243	0.073*	
C1	0.53371 (16)	0.42063 (14)	0.18003 (12)	0.0439 (3)	
C2	0.6380 (2)	0.58510 (16)	0.11281 (15)	0.0590 (4)	
H2	0.6410	0.6752	0.0941	0.071*	
C3	0.74410 (16)	0.37803 (13)	0.12985 (12)	0.0436 (3)	
C4	0.64133 (15)	0.32788 (13)	0.17980 (11)	0.0411 (3)	
C5	0.83458 (16)	0.15897 (14)	0.08849 (12)	0.0443 (3)	
C6	0.70775 (18)	0.11594 (16)	0.00632 (14)	0.0529 (4)	
H6	0.6337	0.1773	−0.0293	0.064*	
C7	0.6902 (2)	−0.01899 (18)	−0.02337 (15)	0.0645 (5)	
H7	0.6035	−0.0482	−0.0777	0.077*	
C8	0.8007 (2)	−0.10947 (17)	0.02737 (17)	0.0659 (5)	
H8	0.7892	−0.1997	0.0070	0.079*	
C9	0.9276 (2)	−0.0667 (2)	0.10786 (17)	0.0731 (5)	
H9	1.0029	−0.1277	0.1416	0.088*	
C10	0.9443 (2)	0.06674 (19)	0.13924 (15)	0.0627 (4)	
H10	1.0302	0.0948	0.1950	0.075*	
C11	0.9716 (2)	0.36428 (19)	0.0848 (2)	0.0854 (7)	
H11A	0.9328	0.3842	0.0061	0.128*	
H11B	1.0556	0.3058	0.0994	0.128*	
H11C	1.0013	0.4462	0.1263	0.128*	

### Atomic displacement parameters (Å^2^)

**Table:**

	*U*^11^	*U*^22^	*U*^33^	*U*^12^	*U*^13^	*U*^23^
Cl1	0.0518 (2)	0.0508 (2)	0.0909 (3)	−0.00514 (16)	0.0415 (2)	−0.00430 (18)
N1	0.0596 (8)	0.0419 (7)	0.0655 (8)	0.0004 (6)	0.0290 (7)	−0.0013 (5)
N2	0.0625 (8)	0.0450 (7)	0.0612 (8)	−0.0097 (6)	0.0322 (7)	−0.0021 (5)
N3	0.0441 (7)	0.0521 (7)	0.0666 (8)	−0.0087 (5)	0.0294 (6)	−0.0103 (6)
N4	0.0722 (9)	0.0451 (7)	0.0856 (10)	0.0066 (6)	0.0541 (9)	0.0102 (6)
C1	0.0441 (7)	0.0418 (7)	0.0507 (8)	−0.0071 (6)	0.0225 (7)	−0.0064 (6)
C2	0.0754 (11)	0.0386 (7)	0.0744 (11)	−0.0032 (7)	0.0400 (10)	0.0019 (7)
C3	0.0439 (7)	0.0441 (7)	0.0461 (7)	−0.0073 (6)	0.0195 (6)	−0.0064 (6)
C4	0.0426 (7)	0.0372 (7)	0.0457 (7)	−0.0068 (5)	0.0180 (6)	−0.0057 (5)
C5	0.0432 (7)	0.0482 (8)	0.0464 (7)	0.0000 (6)	0.0215 (6)	−0.0025 (6)
C6	0.0468 (8)	0.0577 (9)	0.0529 (8)	0.0021 (7)	0.0148 (7)	−0.0053 (7)
C7	0.0640 (11)	0.0676 (11)	0.0659 (11)	−0.0135 (8)	0.0272 (9)	−0.0210 (8)
C8	0.0855 (14)	0.0476 (9)	0.0830 (13)	−0.0023 (9)	0.0525 (12)	−0.0074 (8)
C9	0.0815 (13)	0.0634 (11)	0.0800 (13)	0.0262 (10)	0.0348 (11)	0.0136 (9)
C10	0.0518 (9)	0.0723 (11)	0.0595 (10)	0.0099 (8)	0.0126 (8)	0.0002 (8)
C11	0.0773 (13)	0.0696 (12)	0.140 (2)	−0.0238 (10)	0.0768 (15)	−0.0253 (12)

### Geometric parameters (Å, °)

**Table:**

Cl1—C1	1.7440 (14)	C5—C6	1.377 (2)
N1—C2	1.326 (2)	C5—C10	1.381 (2)
N1—C1	1.3296 (19)	C6—C7	1.389 (2)
N2—C2	1.329 (2)	C6—H6	0.9300
N2—C3	1.3309 (18)	C7—C8	1.374 (3)
N3—C3	1.3876 (18)	C7—H7	0.9300
N3—C5	1.4320 (19)	C8—C9	1.367 (3)
N3—C11	1.467 (2)	C8—H8	0.9300
N4—C4	1.3633 (18)	C9—C10	1.379 (3)
N4—H4A	0.8600	C9—H9	0.9300
N4—H4B	0.8600	C10—H10	0.9300
C1—C4	1.3850 (19)	C11—H11A	0.9600
C2—H2	0.9300	C11—H11B	0.9600
C3—C4	1.4282 (18)	C11—H11C	0.9600
			
C2—N1—C1	114.28 (13)	C10—C5—N3	119.55 (15)
C2—N2—C3	117.64 (12)	C5—C6—C7	120.07 (16)
C3—N3—C5	121.99 (11)	C5—C6—H6	120.0
C3—N3—C11	117.18 (13)	C7—C6—H6	120.0
C5—N3—C11	114.41 (12)	C8—C7—C6	120.14 (17)
C4—N4—H4A	120.0	C8—C7—H7	119.9
C4—N4—H4B	120.0	C6—C7—H7	119.9
H4A—N4—H4B	120.0	C9—C8—C7	119.88 (16)
N1—C1—C4	126.12 (12)	C9—C8—H8	120.1
N1—C1—Cl1	115.43 (10)	C7—C8—H8	120.1
C4—C1—Cl1	118.42 (11)	C8—C9—C10	120.19 (17)
N1—C2—N2	126.91 (14)	C8—C9—H9	119.9
N1—C2—H2	116.5	C10—C9—H9	119.9
N2—C2—H2	116.5	C9—C10—C5	120.57 (18)
N2—C3—N3	117.05 (12)	C9—C10—H10	119.7
N2—C3—C4	121.39 (13)	C5—C10—H10	119.7
N3—C3—C4	121.33 (12)	N3—C11—H11A	109.5
N4—C4—C1	122.70 (12)	N3—C11—H11B	109.5
N4—C4—C3	124.08 (13)	H11A—C11—H11B	109.5
C1—C4—C3	113.19 (12)	N3—C11—H11C	109.5
C6—C5—C10	119.13 (15)	H11A—C11—H11C	109.5
C6—C5—N3	121.28 (14)	H11B—C11—H11C	109.5
			
C2—N1—C1—C4	1.8 (2)	N3—C3—C4—N4	3.9 (2)
C2—N1—C1—Cl1	179.82 (12)	N2—C3—C4—C1	7.6 (2)
C1—N1—C2—N2	3.2 (3)	N3—C3—C4—C1	−178.05 (13)
C3—N2—C2—N1	−2.2 (3)	C3—N3—C5—C6	41.5 (2)
C2—N2—C3—N3	−178.27 (15)	C11—N3—C5—C6	−109.51 (18)
C2—N2—C3—C4	−3.7 (2)	C3—N3—C5—C10	−140.87 (15)
C5—N3—C3—N2	−145.53 (14)	C11—N3—C5—C10	68.1 (2)
C11—N3—C3—N2	4.8 (2)	C10—C5—C6—C7	1.0 (2)
C5—N3—C3—C4	39.9 (2)	N3—C5—C6—C7	178.65 (13)
C11—N3—C3—C4	−169.80 (17)	C5—C6—C7—C8	−1.5 (2)
N1—C1—C4—N4	171.27 (15)	C6—C7—C8—C9	0.5 (3)
Cl1—C1—C4—N4	−6.7 (2)	C7—C8—C9—C10	0.8 (3)
N1—C1—C4—C3	−6.8 (2)	C8—C9—C10—C5	−1.2 (3)
Cl1—C1—C4—C3	175.23 (10)	C6—C5—C10—C9	0.3 (2)
N2—C3—C4—N4	−170.43 (14)	N3—C5—C10—C9	−177.38 (14)

### Hydrogen-bond geometry (Å, °)

**Table:**

*D*—H···*A*	*D*—H	H···*A*	*D*···*A*	*D*—H···*A*
N4—H4A···N1^i^	0.86	2.28	3.0993 (18)	159

Symmetry codes: (i) −*x*+1, *y*−1/2, −*z*+1/2.
